# Homology and Modular Evolution of CATCHR at the Origin of the Eukaryotic Endomembrane System

**DOI:** 10.1093/gbe/evab125

**Published:** 2021-06-01

**Authors:** Carlos Santana-Molina, Fernando Gutierrez, Damien P Devos

**Affiliations:** 1 Centro Andaluz de Biología del Desarrollo, Consejo Superior de Investigaciones Científicas/Universidad Pablo de Olavide/Junta de Andalucía, Carretera de Utrera km 1, Seville, Spain; 2 Departamento de Genética Molecular y Microbiología, Facultad de Ciencias Biológicas, Pontificia Universidad Católica de Chile, Santiago, Chile

**Keywords:** CATCHR, exocyst, COG, GARP, DSL1, membrane trafficking, protein-complex evolution, paralogs

## Abstract

The membrane trafficking is an essential process of eukaryotic cells, as it manages vesicular trafficking toward different parts of the cell. In this process, membrane fusions between vesicles and target membranes are mediated by several factors, including the multisubunit tethering complexes. One type of multisubunit tethering complex, the complexes associated with tethering containing helical rods (CATCHR), encompasses the exocyst, COG, GARP, and DSL1 complexes. The CATCHR share similarities at sequence, structural, and protein-complex organization level although their actual relationship is still poorly understood. In this study, we have re-evaluated CATCHR at different levels, demonstrating that gene duplications followed by neofunctionalization, were key for their origin. Our results, reveals that there are specific homology relationships and parallelism within and between the CATCHR suggesting that most of these complexes are composed by modular tetramers of four different kinds of proteins, three of them having a clear common origin. The extension of CATCHR family occurred concomitantly with the protein family expansions of their molecular partners, such as small GTPases and SNAREs, among others, and likely providing functional specificity. Our results provide novel insights into the structural organization and mechanism of action of CATCHR, with implications for the evolution of the endomembrane system of eukaryotes and promoting CATCHR as ideal candidates to study the evolution of multiprotein complexes.

Significance

The membrane trafficking is an essential feature of the eukaryotic cell managing the movement of molecules toward different subcellular locations. This transport is realized through the vesicular trafficking which is orchestrated by factors including the multisubunit tethering complexes such as CATCHR. We unveil the mode of appearance and diversification of these complexes in eukaryotes concluding that all CATCHR—the exocyst, COG, GARP, and DSL1—are homologs, deriving from one ancestral CATCHR tetramer and presenting modular behavior. This result immediately suggests shared organizational principles and common mechanisms of action of CATCHR. In addition, our results illustrate that the paralogous origin of CATCHR proteins and tetramers was key for the development of the eukaryotic endomembrane system.

## Introduction

The development of the cellular endomembrane system was one of the main triggers of the emergence of eukaryotic life. One essential part of this system is vesicle trafficking, which manages the movement of molecules toward different parts of the cell and requires several processes, namely cargo recognition, coat formation, budding/scission, uncoating, delivery, and fusion. For the latter process, multisubunit tethering complexes (MTCs) are in general terms, mediators of the initial interaction between transport vesicles and their target membranes.

MTCs are large heteromeric complexes that vary in the number and composition of their subunits. They are divided into three main groups with internal relationships, but which are not evolutionarily related between them (Koumandou et al. 2007). The first group comprises the homotypic fusion and vacuole protein sorting (HOPS) complex and the class C core vacuole/endosome tethering (CORVET) complex, which are required for endolysosomal transport. The second group comprises the transport protein particle (TRAPP) complexes, which have a role in transport from the endoplasmic reticulum (ER) to the Golgi acting as a multisubunit nucleotide exchange factor. The third group, complexes associated with tethering containing helical rods (CATCHR), consists of the following complexes: conserved oligomeric Golgi (COG), Golgi-associated retrograde protein (GARP), exocyst, and dependent on Sly1-20 (DSL1). The four CATCHR are widely conserved from plants to humans and also protists (Koumandou et al. 2007). Each one has a specialized function at a particular location in the secretory pathway, including vesicle recycling (Bröcker et al. 2010). DSL1 is involved in Golgi-to-ER transport, GARP is implicated in the recycling pathway from endosomes to the Golgi, COG regulates retrograde transport through the Golgi, and the exocyst coordinates fusion at active sites of secretion in the plasma membrane. Some CATCHR are modular; for example, the endosome-associated recycling protein (EARP) complex is an alternative version of GARP in which the Vps54 subunit is replaced by its homolog, Vps50 (Schindler et al. 2015).

Although GARP is a tetrameric complex, the COG and exocyst complexes consist of one octamer comprising two tetramers (Cog1-4 and Cog5-8, and CorEx1 and CorEx2, respectively). The subunits forming these tetramers, CATCHR proteins, share low sequence similarities with the subunits of other complexes (Whyte and Munro 2001; Koumandou et al. 2007) and some limited structural similarities based on helical bundles arranged in tandem (Dong et al. 2005; Sivaram et al. 2006; Croteau et al. 2009; Vasan et al. 2010). These helical bundles are denoted as domains A, B, C, and D in Exo70 (Dong et al. 2005), with an additional E domain in other proteins such as Tip20, Sec6, Cog4, and Sec10 (Richardson et al. 2009; Tripathi et al. 2009; Chen et al. 2017). In addition to these helical bundles, CATCHR proteins usually have a coiled-coil (CC) region at the N-terminus (Whyte and Munro 2002). Cryo-electron microscopy reconstructions of the exocyst suggest that the CCs are involved in the proper assembly of this complex (Mei et al. 2018). The composition of the DSL1 complex is a notable exception, as only two of its four subunits Dsl1/Zw10 (in fungi and metazoa, respectively) and Tip20 share sequence and structural similarities with other CATCHR proteins (Tripathi et al. 2009). The four proteins of the DSL1 complex are distributed more irregularly than other CATCHR, have strong sequence divergence even between orthologs, and appear to have different functions across the eukaryotic lineages (Spang 2012; Klinger et al. 2013).

The questions of the origin of and the relationship between the CATCHR was posed early and are still open. Some evidence of homology has been reported between CATCHR proteins. However, the presence of the CC region could lead to signal blurring, which led to the suggestion that the CATCHR emerged by convergent evolution of similar secondary structural elements (Koumandou et al. 2007). At that time, the structural characterization of these proteins was limited. Since then, various related structures have been solved, revealing that most CATCHR proteins are structurally similar, which suggests an evolutionary connection (Sivaram et al. 2006; Richardson et al. 2009; Chen et al. 2017). Similarly, the structural organization of CATCHR also has been subject of study (Lees et al. 2010; Chou et al. 2016; Picco et al. 2017; Mei et al. 2018), showing a similar structural conformation between GARP and Cog1-4 (sub-)complexes.

Thus, there is a growing feeling that CATCHR might be related, but evidence is still lacking. Here, we investigated this possibility at the sequence and structural level demonstrating that CATCHR share specific homologies within and between the complexes. By mapping these homologies onto the structural conformation of the complexes we reveal a parallelism between the tetramers forming each CATCHR. Together, we conclude that the homologies between CATCHR proteins, are explained by the duplication and neofunctionalization of an ancestral tetramer with a modular identity. Thus, our results provide novel insights into the complex organization and function of CATCHR and the evolution of the endomembrane system of the eukaryotes.

## Results

### CATCHR Complexes Were Established before Eukaryotic Diversification and Have Evolved Distinctively in Different Organisms

#### Distribution of CATCHR Orthologs across Eukaryotes

We detected no significant hits of CATCHR protein searches against prokaryotic proteomes (even using curated hidden Markov models [HMMs] that were built in this study), thus establishing CATCHR complexes as eukaryotic innovations. We then looked at the conservation of CATCHR proteins across the eukaryotic domain. The identification and classification of CATCHR orthologs are challenging due to their extreme sequence divergence and the existence of diverse paralogs within the CATCHR protein family. Previous analyses have tackled this question by simple reciprocal BLAST (Koumandou et al. 2007) or by reciprocal BLAST in combination with HMM searches and HMM comparisons (Klinger et al 2013). Both analyses started from sequences from *Homo sapiens* and *Saccharomyces cerevisiae*. In this study, we combined two approaches: one involved reciprocal searches of single proteins and the other involved reciprocal searches based on HMM starting from the sequences of *H. sapiens*, *S. cerevisiae*, and *Arabidopsis thaliana* (see Materials and Methods). Then, a consensus profile was derived based on the best e-value hits with reciprocal validations. We use the combination of both because we detect false positives and negatives from HMM approach due to two main reasons. One is the sequence features of these proteins such as coiled-coil (CC) regions whose evolutionary signal can be confusing. In addition, the possible overrepresentation of certain protein families can provoke unspecific HMM models. This was the case of proteins such as Sec20 which are CC proteins belonging to the large protein family of SNARES*.* The second reason is that the automatic realization of HMM can include a mix of orthologs which provokes sub- or overrepresented e-values for the real ortholog assignment. These issues were reduced by using the combination of both, reciprocal searches of single proteins and protein models.

**Fig. 1.**
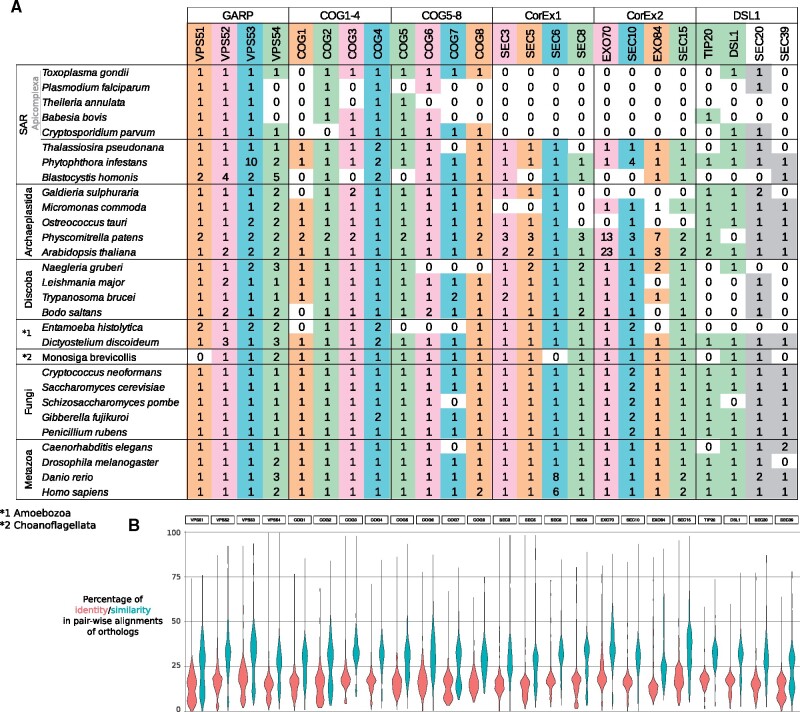
(*A*) Distribution of CATCHR subunits across selected eukaryotes. Numbers indicate the number of co-orthologs for a specific CATCHR protein. Note that some CATCHR orthogroups also include other paralogs, such as Vps50 within the Vps54 group. Note that the detection of orthologs is a combination of our analyses and manual comparisons with other studies and UniProt. Extended information is available in supplementary table 1 and supplementary information data, Supplementary Material online. Columns are colored according to the classification of CATCHR proteins defined in this study. (*B*) Distribution of identity and similarity percentages of pairwise alignments between orthologs.

Our analyses identified orthologs that were not previously detected in literature (Koumandou et al. 2007; Klinger et al. 2013) validating our workflow. This improvement includes the detection of Cog7/Sec20 in *Toxoplasma gondii*, Cog3/Cog5/Cog6/Tip20 in *Babesia bobis*, Vps51/Cog3/Cog5/Cog7/Cog8/Sec20 in *Cryptosporidium parvum*, Vps51/Vps54 in *Dyctyostelium discoideum*, Vps51/Vps54/Cog5 in *Caenorhabditis elegans*. These differences are mainly found when compared with Komandou et al., and this is most likely due to the fact that they employed reciprocal searches of single proteins. Thus, our results show that GARP, exocyst, COG, and DSL1 complexes are conserved in Metazoa, Fungi, Choanoflagellata, Discoba, Archaeplastida, and SAR, although they show irregular distribution in some clades like the absence of exocyst and other CATCHR proteins in Apicomplexa (SAR), the absence of CorEx2 in *Gladieria sulphuraria* (red algae), the absence of DSL1 complex in *Entamoeba histolytica*, and some other punctual absences in other organisms (fig. 1*a* and supplementary table 1, Supplementary Material online). Therefore, in agreement with previous analyses (Koumandou et al. 2007), the broad conservation of these complexes in distant eukaryotes suggests that these complexes were established in the last eukaryotic common ancestor (LECA), and consequently, the absence of CATCHR proteins in some microorganisms can be attributed to secondary losses or extreme sequence divergence. Pairwise alignments between orthologs show low sequence identity and similarity (lower than ∼20% and 30%, respectively; fig. 1*b*) demonstrating the sequence divergence of these proteins in a low range of sequence homology (twilight zone; Rost 1999). This fact highlights the possibility that the absence of CATCHR orthologs in some organisms could be due to important sequence divergence impeding their identification (Boehm et al. 2017).

Some CATCHR protein orthologs present more than one version in different lineages indicating gene duplications of CATCHR proteins (fig. 1*a*). These gene duplications can be ancestral in eukaryotic evolution like Vps50/Vps54 providing a modular identity to the GARP and EARP complexes, respectively (Schindler et al. 2015). Other gene duplications are lineage-specific like Sec10/Rcy1 in fungi, or Sec6/M-Sec (plus others) in vertebrates. Although M-Sec is known to cooperate with exocyst complex (Hase et al. 2009), Rcy1 has not been described to cooperate with its original complex (exocyst) or other CATCHR. Thus, gene duplications followed by neofunctionalization of CATCHR proteins have resulted in important evolutionary innovations by providing CATCHR complexes with modular identity (like Vps50, or M-Sec) or providing proteins working independently of their original complex (like possibly Rcy1).

#### Variation in Domain Architecture of CATCHR Proteins

We then looked at the domain architecture of the detected orthologs, annotating them by secondary structure and Pfam domains (supplementary fig. 1, Supplementary Material online). In addition to cases that appear more stable across the eukaryotes (in terms of size and domain architecture), such as Exo70 (supplementary fig. 1, Supplementary Material online), we observed plasticity of size and domain composition of certain CATCHR proteins. This includes the gain of functional domains, either taxon-specific (as in Sec5 and Exo84; supplementary fig. 1, Supplementary Material online) or basally in eukaryotic evolution (as in sec3-PIP2), as well as the loss of functional domains, such as the shortening of the helical rod body while conserving the region that contains the putative CC (as in Vps51 orthologs; supplementary fig. 1, Supplementary Material online). This plasticity also includes the divergence of functional domains, like the helical rod body, as a basal event in eukaryotic evolution (as in Vps54 and Vps50). Therefore, these four types of protein evolution found in CATCHR proteins—namely the gain, loss, divergence, and conservation of functional domains—suggest that these proteins have been subject to different evolutionary pressures at the molecular level. This in turn indicates that the functional dynamics of CATCHR could vary by taxonomic group.

Another notable observation from our analysis is that the Pfam domains of Vps51, Dor1 (Cog4), Sec5, Vps54_N, Cog2, and Cog5 usually overlap at the N-terminus of CATCHR proteins, especially in Vps51 orthologs (supplementary fig. 1, Supplementary Material online). These Pfam domains are mapped onto the predicted CC regions, revealing a common feature between CATCHR proteins and corroborating the recognized confounding effect associated with CCs in sequence analyses (Mistry et al. 2013). This also reveals that in some cases, the Pfam domains defining CATCHR proteins can be unspecific, particularly in the case of CC fragments.

### Evolutionary Relationships between CATCHR Proteins Define Diverse and Coherent Classes

#### Relationships Based on Sequence Similarity

The similarities previously reported between CATCHR proteins could be due to sequence convergence in the CC regions (Koumandou et al. 2007), although the increase in structural information available for these proteins is challenging this view (Richardson et al. 2009). We further investigated this issue by analyzing the homology between the proteins based on HMM comparisons using different approaches: one with an automated workflow for the generation of HMM and the other with HMMs generated using the orthologs detected in this study (see Materials and Methods). Although the first method could include a mix of orthologs in the production of HMM with the possibility to detect more remote homology, the second avoid this issue and uses a taxonomically balanced data set.

**Fig. 2.**
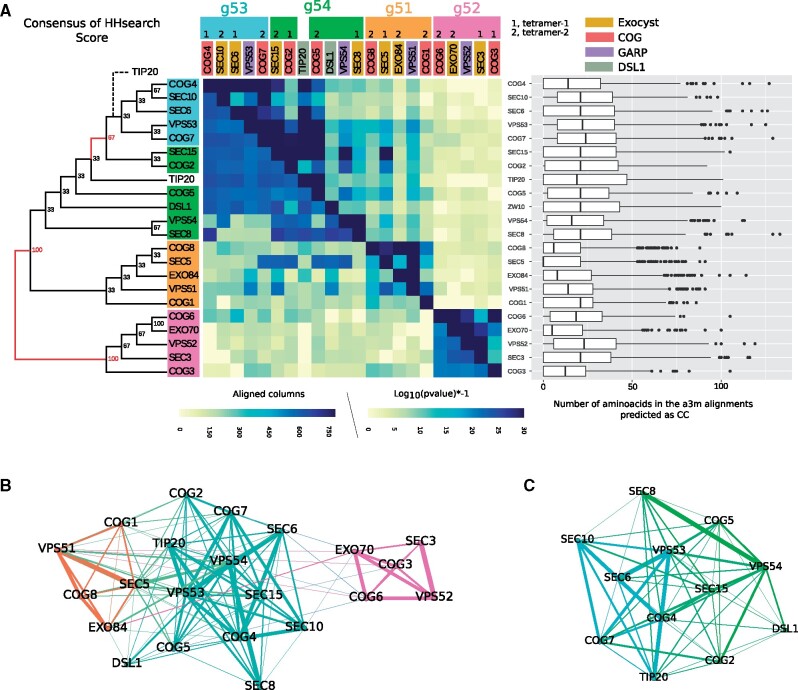
Relationships of CATCHR subunits based on their protein sequence. (*A*) Comparison of Hidden-Markov models (HMM) built automatically. The cladogram is the consensus of the relationship between the HMMs of CATCHR proteins as described in Materials and Methods section. Labels are colored according to the congruence (>66%) between the three analyses. The asymmetrical heatmap shows the length of the alignment above the diagonal, and the log_10_ of the *P* value of hits from the *Homo sapiens* analysis below the diagonal. Note that *P* value scale was limited up to 1e-20 as maximum to ease the visualization of lower this. The boxplots show the number of amino acid positions that are predicted to form coiled-coil regions. (*B*) Clustering network of HMM comparisons between CATCHR proteins without considering the coiled-coil region. The HMM were built using the the sequences obtained from the orthology analyses. (*C*) Clustering network of g53 (cyan) and g54 (green) clusters defined by modularity. Raw data of these analyses are provided in supplementary information data, Supplementary Material online.

For the automated approach, we performed a hierarchical clustering based on the scores of the HMM comparisons built from automated searches. These HMM were built starting from the sequences of *H. sapiens*, *S. cerevisiae*, and *A. thaliana*, respectively. We build a consensus-based cladogram obtained from the hierarchical clustering from the three analyses. We defined four clusters of proteins whose relationships are replicated in at least two of the three analyses (>66% of congruence; fig. 2*a*) although two of these clusters show some overlap. As GARP is formed by only four CATCHR proteins, with one in each potential cluster, we named these clusters according to the GARP subunit that they contained: g51, g52, and g53-g54. The coverage of the alignments behind the formation of these clusters is higher than the total number of amino acids predicted to form CC indicating that the sequence similarities between proteins in the same cluster are not limited to the CC region but rather extend beyond it (fig. 2*a*).

We performed a second approach to confirm and complement the definition of these clusters. We realized a clustering network of HMM comparisons whose protein models were assembled from the orthology analyses providing a taxonomically balanced set of sequences. The HMMs were built including and excluding the CC regions (see Materials and Methods) and both analyses provided similar results (fig. 2*b* and *c* and supplementary fig. 2, Supplementary Material online), corroborating that the clustering is not solely due to the CC regions. The clustering network considered all hits below 1e-2 of *P* value threshold and resulted in three clusters, corresponding to the previous ones: g51, g52, and g53-g54 (fig. 2*b*). However, a clustering network considering only the subset g53-g54 obtained a separation between these two clusters (fig. 2*c*), supporting the existence of these two clusters obtained in the first approach, and suggesting that their separation is blurred by the other clusters. On the other hand, we noticed that Tip20 shifted to the g53 cluster (in contrast to the first approach, fig. 2*a*). This happened because in the first approach Tip20 and Cog5 had similarity, but in the second approach, there is no similarity detected between these two proteins. This is likely due to the mix of orthologs in the generation of HMM in the automatic approach. Thus, due to the possible limitation of the first approach, Tip20 will from here be considered as a member of g53. Therefore, despite this difference, both approaches provide congruent results supporting the establishment of the four clusters of CATCHR proteins, although it is worth mentioning that the definition of g53 and g54 clusters is unstable. The instability of both groups is explained by the irregular sequence conservation between their members. For example, Sec15 and Cog2 (from g54) have stronger similarities with some g53 proteins than with others from its own group. By contrast, Dsl1, Vps54, and Sec8 (also from g54) have lower similarities with g53 but also between themselves (compared with the higher similarities between g53 proteins; fig. 2*a*). Indeed, Dsl1/Zw10 is the CATCHR protein with the lowest similarity scores with other proteins, that is, the most divergent CATCHR protein. On the other hand, we think that the instability of these two groups is an intrinsic feature of the data due to the possible functional speciation of CATCHR proteins in a low range of sequence homology. Thus, despite these irregularities in the sequence conservation between g53 and g54, our results support the existence of both clusters which are coherent with the structural organization of these tetrameric complexes (see below).

One notable outcome of these analyses is that each cluster contains one protein from each tetrameric CATCHR (GARP, CorEx1, CorEx2, Cog1-4, Cog5-8, or one of the two DSL1 subunits; fig. 2). Thus, the g51 cluster contains Vps51, Cog1, Cog8, Sec5, and Exo84; the g52 cluster is formed by Vps52, Cog3, Cog6, Sec3, and Exo70; the g53 cluster contains Vps53, Cog4, Cog7, Sec6, Sec10, and Tip20; and the g54 cluster contains Vps54, Cog2, Cog5, Sec8, Sec15, and Dsl1/Zw10. Hence, the homology between the proteins reveals a coherent relationship between all the CATCHR, reflecting the relationship at the complex and subcomplex (tetramer) level.

Several conclusions can be derived from this analysis. First, the g53 and g54 clusters originated by gene duplication, as the *P* value and coverage of the alignments reveal homology between the members of these two clusters (fig. 2*a*). Second, the g51 cluster is related to g53 and g54, although more remotely. Third, the g52 cluster is less related to the other three clusters and contains the proteins described as membrane-anchoring components of CATCHR, such as Exo70 and Sec3 (He and Guo 2009; Liu et al. 2018). This function could probably be extended to most members of g52, as previously suggested (Whyte and Munro 2001). Fourth, the hits of HMM comparisons between members of g52 and the other three clusters showed very low sequence similarities (fig. 2). We inspected these hits by looking at the distribution of their cover alignment and e-values (supplementary fig. 3, Supplementary Material online). We observed that g52 alignments with the g51, g53, and g54 protein models mainly encompass the first 300 positions, and even ∼500 in some cases, such as Cog3 and Cog6 (supplementary fig. 3*a*, Supplementary Material online), indicating that the alignments between g52 and the others can extend beyond the CC. However, these similarities are weak as the e-value of such hits were mainly in the order of 1e + 2 (supplementary fig. 3*b*, Supplementary Material online). By contrast, the comparisons of g51, g53, g54 with each other (but not with themselves) showed longer alignments (>600 positions) with a main distribution of e-values in the order <1 (Supplementary fig. 3, Supplementary Material online). Thus, we detected strong evidence of homology between g53 and g54 and, albeit more weakly, g51. Conversely, g52 share weak sequence similarities with the three others which are congruent as they map at the N-region and that in some cases extends beyond the CC region.

Regarding the relationship between the complexes, we performed a clustering network considering only those four first hits with and *P* value lower than 1e-2 and coverage of the alignment higher than 300 positions (HMM from otrhologous data set; supplementary fig. 2*c*, Supplementary Material online). We observe that proteins from the same complex and the same cluster, do not present higher similarities. Instead, the GARP proteins have higher similarity scores with proteins of the exocyst and in particular with the CorEx1. Similarly, the strongest similarities of Dsl1 and Tip20 were with Vps54 and Vps53, respectively (from the GARP). On the other hand, COG subunits showed higher similarity scores with proteins from the GARP and exocyst complexes.

Altogether, these results reflect the sequence similarities that have been reported since the identification and characterization of these proteins (Whyte and Munro 2001; Koumandou et al. 2007). They reveal that there are specific homologies within and between the CATCHR that extend beyond the CC region, which provides the first comprehensive evidence of direct relationships between the different CATCHR. These homologies suggest that gene duplications followed by neofunctionalization played a key role in the emergence of these complexes.

#### Relationship Based on Structural Similarity

To further characterize the evolutionary relationships between the CATCHR proteins, we analyzed some of their sequence and structural features. We aligned the sequences from each group of orthologs (fig. 1) and then aligned the alignments within each cluster. The resulting alignments show conserved positions enriched in hydrophobic amino acids (supplementary fig. 4, Supplementary Material online), suggesting a possible pattern of hydrophobic residues involved in the packing of the helical bundles. Moreover, despite poor sequence conservation even between orthologs, the alignments display specific motifs of charged and polar amino acids conserved across the entire alignment (supplementary fig. 4, Supplementary Material online).

**Fig. 3.**
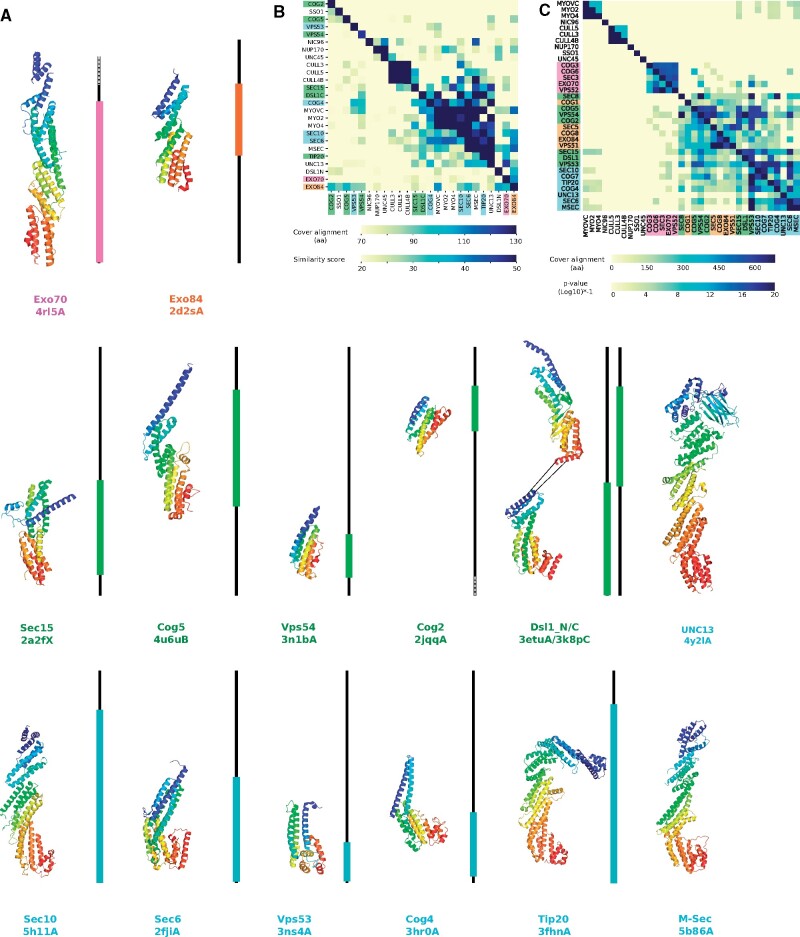
Structures of CATCHR proteins and their relationships. (*A*) Representative PDB chain structures of CATCHR proteins mapped to the length of the multiple sequence alignments (MSAs) of the respective CATCHR cluster as a black line. Colored blocks cover the fragment of the MSA mapped by the structure. PDB chains are colored from N-terminus (blue) to C-terminus (red) and grouped according to the CATCHR clusters defined in this analysis. See extended version of the multiple sequence alignment in supplementary figure 4 and supplementary information data, Supplementary Material online. PDB codes and chains are indicated below the protein names. (*B*) All-versus-all comparison between CATCHR and non-CATCHR protein structures. The asymmetrical heatmap represents the length of the alignments above the diagonal and the respective MOMA structural similarity scores below the diagonal. The labels are sorted according to the average of the SR. (*C*) All-versus-all comparison of the HMMs of CATCHR and non-CATCHR protein sequences. The asymmetrical heatmap shows the length of the alignment above the diagonal as well as the *P* value of the respective HHsearch hits below the diagonal. The CATCHR HMMs were built with the sequences obtained from the orthology analyses, whereas the non-CATCHR HMMs were obtained from the PDB database. The labels are sorted according to the average of the score of HHsearch hits.

Next, we mapped representative protein structures on the multiple sequence alignments (MSAs) of the CATCHR protein orthologous groups (fig. 3*a*). Structural information covering more than half of the alignment is limited but available for each group. Only one structure has been solved for two of the clusters: Exo84 and Exo70 for g51 and g52, respectively. Various structures are available for g54, with whole or partial structures for all kinds of ortholog except Sec8. Dsl1 has been crystallized in two parts, with one structure covering the N-terminal region (from *S. cerevisiae*) and the other covering the C-terminal region (from *Kluyveromyces lactis*), as it contains a flexible fragment involved in the interaction with other molecular partners (Ren et al. 2009). Complete or partial structures are available for all members of g53 except Cog7. Most of these structures correspond to the C-terminal fragment of the proteins, except for the Sec10 structure, which is almost complete (fig. 3*a*). There is thus a bias toward structures of the C-terminal regions, which suggests that the N-termini of these proteins could harbor features hindering crystallization, such as structural flexibility.

We then compared the structures of the CATCHR proteins using a method for flexible structural alignment that was specifically designed to detect remote structural homology (Gutiérrez et al. 2016). We also included the structures of other proteins that have been reported to be structurally related to CATCHR proteins, such as M-Sec (Sec6 co-ortholog), UNC-13, Cullin, MYO, SSO (Chen et al. 2017; fig. 3*a*), and other all alpha-structures as negative controls (UNC45 and Nup170). The resulting all-versus-all comparisons display a mixed clustering between CATCHR and non-CATCHR proteins (fig. 3*b*). We report significant structural similarities between CATCHR proteins and some proteins that share sequence homology, such as M-Sec and UNC13, as well as other proteins that share no significant or apparent sequence homology based on HMM comparisons, such as crystallized regions of SSO, MYO, and Cullin proteins (fig. 3*b* and *c*). Although the former group clearly represents proteins originally derived from CATCHR proteins, the relationship to the latter group is less obvious.

**Fig. 4.**
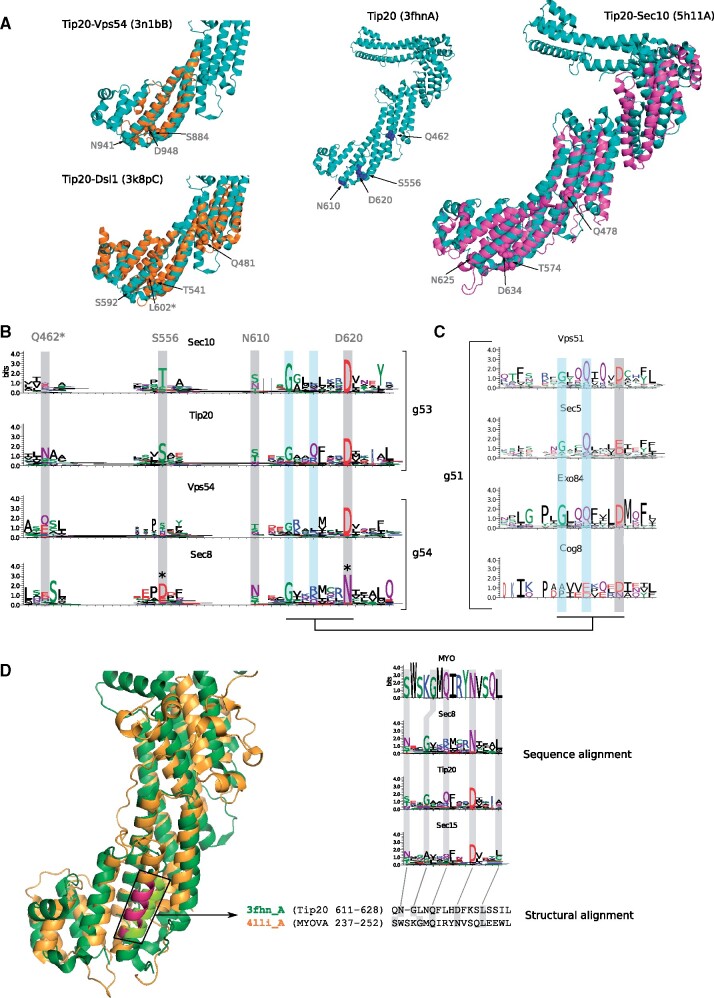
Structural and sequence alignment congruence. (*A*) Structural alignment of Tip20 (green, 3fhnA) and other g53 protein like Sec10 (pink, PDB code: 5h11A) and g54 proteins like Vps54 and Dsl1 (orange, 3n1bA and 3k8pC). The central panel shows the conserved amino acid positions of Tip20 included in the domains C–D. (*B*) Sequence alignment of two representative proteins from the g53 and g54 clusters. The four amino acids shown in the structural alignment are highlighted by a gray background. The asterisk indicates the position of a putative compensatory mutation. (*C*) Sequence alignment of g51 proteins reveals a motif of three residues suggesting that domain C could be present, similar to that seen in g54 and g53 proteins. Positions highlighted in cyan indicate conserved amino acids between g51 and g53–g54 proteins. (*D*) Structural alignment (left panel) of Tip20 (green) and MyoVA (orange) and sequence alignment (right panel) of MyoVA and selected g54 proteins. The alpha helix in dark red corresponds to the region of the alignment shown.

Our structural comparisons reveal higher similarities between the structures of CATCHR proteins from the same cluster than between proteins belonging to different clusters (specially for g53 and g54, which are also the one with more structures solved, fig. 3*a*), supporting our sequence-based clustering. The helical bundles of the CATCHR proteins are denoted A, B, C, and D for g52 and g51 proteins (Dong et al. 2005), and also E for g53 and g54 proteins (Tripathi et al. 2009). The structures of some proteins in g53 and g54 have high structural similarity, including within domains C–D–E. As this region has been proposed to be an ancestral feature of CATCHR proteins (Richardson et al. 2009), we inspected it by mapping the positions of the conserved amino acids in the MSA on the structural alignments. Conserved distal amino acid positions encompassing domains C–D are detected between Tip20/Sec10 and Vps54/Dsl1, representative structures from the g53 and g54 clusters, respectively. These amino acids are equivalently aligned in the sequence- and structure-based alignments (fig. 4*a* and *b* and supplementary fig. 5, Supplementary Material online), which supports the previously suggested homology between these two clusters. Moreover, we observed a possible compensatory mutation between Tip20 and Sec8, in which the structurally adjacent residues S557/D620 in the former are changed to D557/N620 in the latter (fig. 4*a* and *b*), which is suggestive of coevolving sites (de Juan et al. 2013). By contrast and as previously noted (Richardson et al. 2009), the domain E has very low sequence conservation in structural and sequence alignments, even between proteins from the same cluster (supplementary information data, Supplementary Material online). This domain E is not included in the solved structures of some g54 proteins, such as Sec15 or Vps54, although it is expected to be present based on sequence information (fig. 3*a*). Similarly, domain D (and E) is not included in the solved structure of Exo84 from *S. cerevisiae*, the only available structure for g51 proteins, although sequence alignment suggests that it could be present in g51 proteins from other organisms (fig. 4*c*), which agrees with our detection of domain variation in Exo84 orthologs (supplementary fig. 1, Supplementary Material online). Thus, despite the low sequence similarity detected in previous analyses (fig. 2), the presence of domain D in proteins from the g51 cluster (fig. 4*c*) supports the homology between the g51 and g53–g54 proteins. By contrast, this domain D does not align well in the sequence alignment between g51–g53–g54 and g52 (i.e., it forces the introduction of a gap in the alignment), suggesting that the C-termini of g52 proteins are different from the C-termini of other CATCHR proteins. This observation is supported by the poor structural similarities between g52 and g54–g53 protein structures at the C-terminus (Exo70 and Tip20, respectively; supplementary fig. 6*a*, Supplementary Material online). Therefore, the C–D domains of g52 proteins differ from the C–D(–E) domains of g51–g53–g54 proteins.

On the other hand, we observed no structural similarity between Cog5 or Cog2 and other CATCHR proteins, despite their sequence similarity (fig. 4*a* and *b*). However, the associated structures correspond to the N-termini of these proteins, whereas most of the CATCHR structures correspond to the C-termini (fig. 3*a*). This finding suggests that the helical bundles of the N-termini of CATCHR proteins are structurally more variable despite their global sequence similarity. Due to the different behavior detected for the N- and C-termini, we analyzed the structural similarity by temini, N- or C- (supplementary fig. 5*b*, Supplementary Material online). The structure of Exo84 corresponds to the N-terminus of the g51 proteins and superimpose well with the N-termini of Exo70N (the only structure available for the g52; supplementary fig. 6*b* and *c*, Supplementary Material online) which agrees with previous observations by Dong et al (Dong et al. 2005). This is also in agreement with the weak sequence signal that we found between g52 proteins and other CATCHR proteins that could extend beyond the CC (fig. 2 and supplementary fig. 3*a*, Supplementary Material online). Thus, g51 proteins share sequence similarities at the C-terminus with proteins from the g53 and g54 clusters, as well as structural similarity at the N-terminus with some g52 proteins, which is supportive of an evolutionary relationship between g52 and the other CATCHR proteins.

Beyond the CATCHR subunits, we also inspected those proteins that show structural similarity despite low or no sequence similarity, such as Myo or Cullin proteins. Myo proteins and some g54 and g53 proteins have strong structural similarities as they share domains C–D–E (fig. 4*d*). This is in agreement with our finding that the HMM of Myo proteins shows a weak sequence similarity (e-value > 1) of ∼200 amino acids length at the C-terminus with some g54 and g53 proteins (fig. 3*c*). In addition, when we aligned the sequence alignments of g53–54 and Myo proteins, we observed again congruence between the sequence and structural alignments (fig. 4*d*). Thus, this structural and sequence evidence relates Myo and CATCHR proteins, in particular with those from the g53 and g54 clusters, and is indicative of homology. Similarly, although we did not detect any sequence similarity between Cullin and CATCHR proteins, some CATCHR proteins such as Exo84 and Exo70 overlap with the *Cullin_repeat-like_dom_sf* domain, according to the InterPro annotations.

Therefore, taking into account the lack of representative structures of CATCHR proteins, the structural similarities observed here are consistent with and complementary to the results of our sequence-based comparisons (figs. 2–4). In addition, the homology between CATCHR and the CATCHR-like region of MYO proteins suggest strong sequence divergence and functional extension of CATCHR protein family.

## Discussion

Given the presence of CATCHR in the LECA (Koumandou et al. 2007 and this study), we evaluated the homology relationships between their components. We detected homology based on sequence and structure and demonstrated that the sequence similarity extends beyond the CC regions indicating that our relationships are not biased by the low complexity of these fragments. We then classified the CATCHR components into four clusters which are composed of homologous proteins. We show that CATCHR proteins from the g53, g54, and g51 clusters have a common origin, although g51 proteins are more divergent. There is not a strong definition of g53 and g54 clusters probably because these proteins have diverged distinctively which provided irregular similarities between them and blurred the definition of both clusters. A common origin for g52 proteins is not so evident although the weak sequence similarities detected in the N-region together with the high structural similarity of Exo84 with Exo70 suggest an evolutionary relationship including the CC and the helical bundle A. This strong sequence divergence of g52 proteins might be linked to the fact that most of them are known to function as membrane anchors (He and Guo 2009). Each cluster contains one protein from each tetramer suggesting that there was an ancestral tetramer formed by one protein from each cluster (g51, g52, g53, and g54) and that the current CATCHR are the result of consecutive duplication events from an ancestral CATCHR tetramer. The DSL1 complex is the exception which appears to form a half tetramer, in agreement with the peculiar features of this complex like its dual role in kinetochore and membrane trafficking presenting different protein-complex organization in mammalians cells (Tagaya et al. 2014). Therefore, our analyses reveal that CATCHR share specific inter- and intrahomologies, which argues against the view that these complexes emerged by convergent evolution from independent origins (Koumandou et al. 2007).

**Fig. 5.**
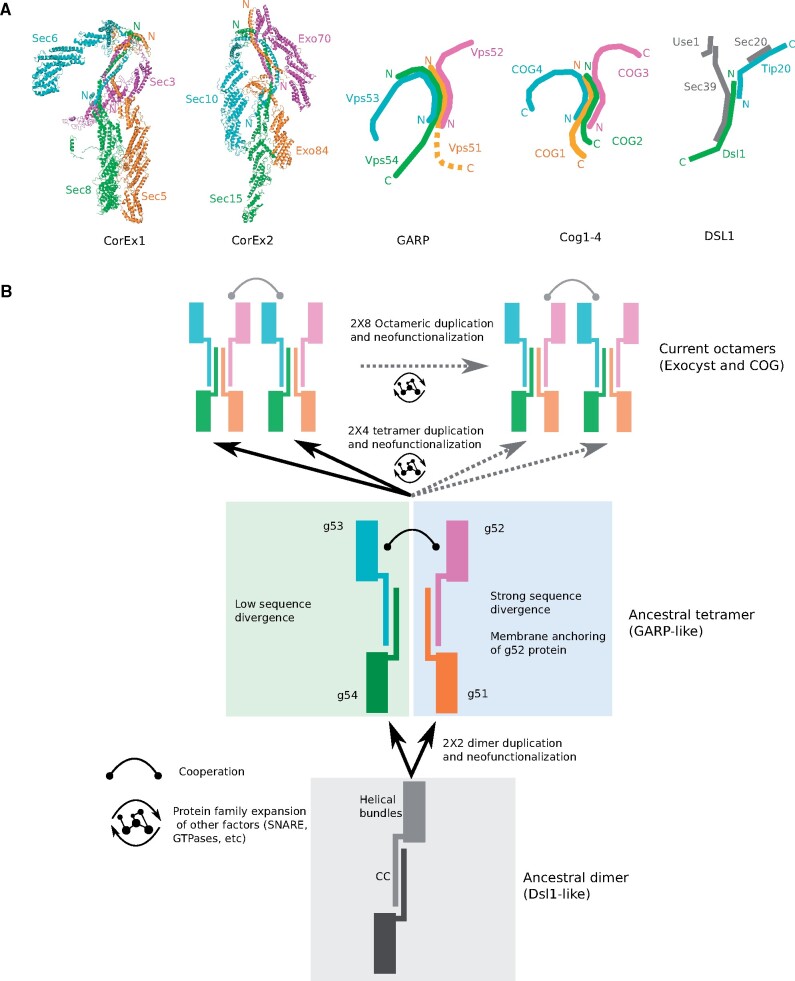
Structural organization of CATCHR complexes and proposed model of origin and evolution. (*A*) Structural conformation of CATCHR tetramers (Chou et al. 2016; Mei et al. 2018; Travis et al. 2020). Subunits are colored according to their CATCHR cluster: g51, orange; g52, pink; g53, cyan; and g54, green. (*B*) Evolutionary model for the origin and development of CATCHR complexes. Note that GARP and DSL1 complexes might be interpreted as representing ancestral states but not necessarily ancestral complexes. Discontinuous lines indicate alternative paths for the emergence of octameric CATCHR.

We then leveraged the structural knowledge of these complexes. GARP and Cog1-4 tetramers display a “Y” shape (Chou et al. 2016) and we found that the CorEx1 and CorEx2 tetramers of the exocyst (Mei et al. 2018) adopt a similar conformation (fig. 5*a*). We mapped our classification of CATCHR proteins onto these tetramers and observed a clear parallelism between the conformations of the four tetramers (fig. 5*a*). The central body of the tetramer is formed by g51 and g54 proteins, whereas the two arms are formed by g52 and g53 proteins. In addition, the N-terminus/C-terminus disposition of the corresponding proteins is equivalent in the four tetramers, characterized by an antiparallel assembly of the CC regions of the g53–g54 and g51–g52 pairs of proteins (Mei et al. 2018). This parallelism between the tetramers of different CATCHR suggests that CATCHR have a modular identity and agrees with our proposal of an ancestral CATCHR tetramer with such a Y shape composed by ancestral g51–g52–g53–g54 proteins (fig. 5*b*). A related Y shape has also been described for the conformation of the DSL1 complex in *S. cerevisiae* although its composition is quite different (Travis et al. 2020; fig. 5*a*). CATCHR proteins of DSL1, Dsl1, and Tip20, represent the g53–g54 half of a CATCHR tetramer illustrating its alternative evolutionary path regarding the other CATCHR and not including the membrane anchoring feature provided by g52 proteins.

The origin of the first ancestral CATCHR tetramer probably involved gene duplication with subsequent neofunctionalization, as is most obvious for the proteins from the g53, g54, and g51 clusters. The g52 proteins are the least closely related, although they appear to share some structural similarities at the N-termini regions. Given that these proteins multimerize by their CC regions (Mei et al. 2018), we hypothesize that the ancestral form of this tetramer was a dimer composed of one protein forming the central body and another forming the arm of the Y shape (fig. 5*b*). Our results show that one of these dimers diverged less (g53–g54) than the other (g51–g52), possibly due to the speciation of the membrane-anchoring function of g52 proteins. Indeed, the existence of an ancestral dimer is supported by the fact that DSL1 can act as a dimer in cooperation with different proteins (fig. 5*a*). Thus, the origin of the first ancestral tetramer was most likely determined by the functional success of the cooperation of two dimers. Likewise, once this ancestral tetramer was formed, the duplication of the tetramers involved the subsequent duplication of the entire complexes, acquiring novel functionalities as well as more precise subcellular localization. Therefore, duplication and new neofunctionalization in the CATCHR family have happened at two levels: at the protein level, for the origin of the first ancestral tetramer; and at the tetramer level, for the different complexes. CATCHR proteins share related interactors, including SNAREs and small GTPases (Ras superfamily), which suggests that the protein family expansions extended beyond the complexes to their interactors (Bröcker et al. 2010). For example, small GTPases such as Rho/Ras subfamily are expected to act at the plasma membrane and interact with exocyst proteins (Mukherjee et al. 2014). Conversely, GARP interact with Arf small GTP-ases such as Arl1 regulating the dynamics of the Golgi (Yu and Lee 2017). Similarly, various Rab proteins also regulate the functions of COG complex in the dynamics of the Golgi (Willett et al. 2013). However, it is also possible that small GTPases from the same subfamily interact with different CATCHR (Bröcker et al. 2010). Therefore, it seems that each CATCHR has its own set of small GTPases that defines its subcellular location and similar observations are given for other protein families such as SNARE (Koumandou et al. 2007; Bröcker et al. 2010). Together, this view reconciles with the organelle paralogy hypothesis, which proposes that the increase in complexity was caused by iterative gene duplications, followed by sequence divergence and neofunctionalization in multiple interacting proteins encoding organelle identity and pathway specificity (Dacks and Field 2007).

These observations raise the question of the genomic mechanisms behind the expansions of CATCHR complexes (and possibly other multimeric proteins). We contemplate three main possibilities. The first possibility is independent duplications of CATCHR genes. However, we think that this mechanism more plausibly explains the modular identity of CATCHR than the emergence of an entire complex. The second one is tandem duplications of CATCHR genes. Synteny analyses of different CATCHR genes in different clades did not reveal any specific genomic association between CATCHR genes. However, since the formation of the ancestral tetramer could require tandem duplications (as it is more evident for g51, g53, and g54 genes), it could be expected that the ancestral tetramer had a clustered gene organization in lineages preceding the LECA. Therefore, tandem duplications for exapansion of CATCHR family cannot be discarded. The third possibility (not mutually exclusive with the second one) is whole-genome duplication in organisms preceding the LECA. This possibility is in agreement with the extensive and synchronous protein family exapansions of other eukaryotic components of the endomembrane system and other molecular systems during eukaryogenesis such as Nano/Miss12 complexes of the kinetochore (Tromer et al. 2019), the Sm/Lsm protein families of the spliceosome (Veretnik et al. 2009) or the membrane coat protein family involved in the formation of cell vesicles as well as nuclear pore complex (Devos et al. 2006). Therefore, our study reinforces the idea that gene duplication of certain protein families promotes the emergence of multiprotein complexes.

The order of appearance of CATCHR, could shed light on the order of appearance of each subcellular location in the eukaryotic cell. Indeed, the order of appearance of CATCHR complexes could be reminiscent (and perhaps concomitant) of the evolution of other complexes involved in the vesicular trafficking like adaptor protein complexes forming the coated vesicles (Duden et al. 1991; Schledzewski et al. 1999). Based on our results, it is difficult to decipher the exact order of appearance of all CATCHR probably due to the limitations of HMM comparison in combination with such divergent proteins whose evolutionary signal is eroded in the twilight zone of homology. However, some hypothesis can be speculated based on our results. Since GARP is a single tetramer and the most conserved, this complex may be the closest to the ancestral CATCHR complex. Three additional pieces of evidence agree with this possibility: the overlapping of multiple CATCHR-related Pfam domains at the N-terminus of Vps51 orthologs (supplementary fig. 1, Supplementary Material online), the stronger similarity of GARP proteins with other CATCHR proteins (supplementary fig. 2*c*, Supplementary Material online), and the ancestral modular behavior of GARP/EARP and its early establishment before the LECA (Schindler et al. 2015). Thus, if the origin of CATCHR was a single tetramer (like GARP), then, the origin of octameric CATCHR (like COG and the exocyst) was due to the functional success of the cooperation between two duplicated tetramers (fig. 5*b*). Subcomplexes of the exocyst, that is, CorEx1 and CorEx2 apparently do not originate one from the other, and a similar observation is inferred for COG subcomplexes (supplementary fig. 2*c*, Supplementary Material online). However, this view should be interpreted cautiously due to the low sequence conservation and the possible fast evolution after duplication for the neofunctionalization of the tetramers. GARP proteins have the strongest similarities with the exocyst (particularly with the CorEx1 tetramer) which could be suggestive of a direct evolutionary connection between the exocyst and GARP. Although the exocyst complex is involved in vesicle trafficking from the Golgi to the plasma membrane, GARP is involved in transport from endosomes to the Golgi (GARP) which suggests an interesting scenario of an ancestral reverse flow of vesicle trafficking based only on CATCHR systems. On the other hand, the DSL1 complex followed a different evolutionary path as it is composed of a half tetramer of CATCHR and whose CATCHR proteins have again stronger similarities with GARP proteins. DSL1 is known to form different complexes which participate in vesicle trafficking (Dsl1-Tip20-Sec20-Sec39 in *S. cerevisiae* and Zw10-Rint1-Nag [NRZ] in *H. sapiens* which are Dsl1, Tip20, and Sec39 orthologs, respectively) as well as in the kinetochore (Rod-Zwilch-Zw10, RZZ complex; Tagaya et al. 2014). Although Sec20 belong to the family of SNARES such as Stx, Vmp, or Snp, Sec39 (Nag in human) has similarities with proteins related to centriole and microtubule assembly dynamics (like Eml and Poc protein subfamilies) but also with the Rod protein of RZZ complex of the kinetochore (Tromer et al. 2019). Thus, DSL1 represents a versatile complex whose interactors belong to other protein families that have been expanded and which have provided functional specificity to the complex. DSL1 illustrates the diverse modular behavior of these complexes which raises the possibility that alternative CATCHR-based systems remain to be discovered.

In conclusion, by combining sequence and structural information, we have established coherent relationships within and between CATCHR, demonstrating that gene duplication played a key role for the origin of these complexes. We infer that CATCHR comprise proteins with a common origin (g51, g53, and g54) and membrane-anchored proteins (g52) whose homology with the other clusters is less obvious but existing. Mapping the homology relationships onto the structural conformation of CATCHR illustrates a clear parallelism between the tetramers forming them, revealing a modular identity of these complexes. This information is useful for further understanding the conformations of the entire CATCHR as it suggests a similar mechanism of action. Similarly, the modular identity of CATCHR will help to predict and extrapolate the function of each CATCHR subunit. For example, based on our results, it could be hypothesized that the Cog5-8 tetramer has a conformation similar to the one of other CATCHR tetramers. Furthermore, we propose that CATCHR are ideal well-studied models to further study multiprotein complexes evolution. The homology and expansion of CATCHR family extend the organelle paralogy hypothesis for the emergence of the eukaryotic endomembrane system.

## Materials and Methods

### Ortholog Detection and Annotation

The detection of orthologs was carried out by a combination of reciprocal iterative searches of HMM and single proteins (supplementary fig. 7*a*, Supplementary Material online) using the HMMER package (Potter et al. 2018). Analyses were started from three initial sequences: the CATCHR subunits from *S. cerevisiae*, *H. sapiens*, and *A. thaliana*. The reciprocal searches of HMM consisted of four steps for each protein. For the initial search, Jackhmmer searches were performed against UniRef90 2016 release (http://www.uniprot.org), three iterations using 1e-5 as the e-value threshold, generating a HMM of each protein, and the newly built HMM was used with Hmmsearch (1e-3 as e-value threshold) to search against the selected target proteomes. In this forward search we only considered the best hit for each protein avoiding overcounting of the same orthologue; For the reciprocal search, Jackhmmer was again used to generate a HMM of each hit of the target proteome (four iterations and 1e-2 as the e-value threshold), and Hmmsearch of each HMM against the initial proteome (without e-value threshold) and check if the first hit coincides with the initial query. We evaluated different combinations of e-value thresholds followed by manual inspection of orthologs. We combined the result obtained from the three analyses and considered the best e-value hit to be the correct ortholog assignment. Due to the possible limitation of HMM usage, that is, overrepresented protein families and mix of orthologs for the construction of HMM, we combined the analyses with reciprocal searches of single proteins. These reciprocal searches were carried out with phmmer, with an e-value threshold of 1e-2 for the initial search and 1e-3 for the reciprocal search and with alignment coverage >40%. We compared the result of both approaches by manual inspection and removed false positives. This last step was crucial due to the aforementioned issues and the use of low e-value thresholds in the searches. In addition to these analyses, we further inspected the absences interrogating their possible existence at online databases like UniProt and by comparing with other studies (Koumandou et al. 2007; Klinger et al. 2013). The raw data of the reciprocal searches of HMM and single proteins are provided in supplementary information data, Supplementary Material online.

Once the orthologs were identified, we used Foundation (Bordin et al. 2018) to identify structurally disordered regions, transmembrane helices, and secondary st*ructur*es. Hmmscan (using the -cut_ga option) was used to identify the domain architecture based on the Pfam database (Finn et al. 2014). To obtain the MSA of the four CATCHR clusters, we first aligned the sequences of each orthogroup using MAFFT-linsi (Katoh and Standley 2013). Then, for each cluster, we aligned these alignments of CATCH*R or*thologs using MAFFT-linsi (-add option). Those regions with more than 80% of gaps were removed using trimAL (Capella-Gutiérrez et al. 2009).

### Homology Detection of CATCHR Proteins

We performed HMMs comparisons to detect homology between CATCHR proteins. We performed two approaches with different methods to build the HMMs and the clustering (supplementary fig. 7*b*, Supplementary Material online). For the first approach, we automatically built a HMM of each protein in the CATCHR complexes (exocyst, COG, GARP, and DSL1) from *H. sapiens, S. cerevisiae*, and *A. thaliana* using the HH-suite tools (Steinegger et al. 2019)*.* To do so, we performed iterative searches for each protein using HHblits with two iterations and no e-value threshold, to generate the corresponding MSA. These MSAs were used to build a HMM of each CATCHR protein through HHmake. We compared the HMMs of CATCHR proteins for each organism. We used the PDB-HMM database as background and added our HMMs. Then, we performed HMM searches of each HMM of the respective organism using HHsearch. We made a comparative matrix of the score of the HHsearch build a cladogram of CATCHR proteins for each organism applying a hierarchical clustering based on the average of the values (using python *SciPy* packages). Finally, we built a consensus using these three cladograms and as example, we also show the length of the HMM alignments and the *P* value of the respective hits for the human proteins (fig. 2*a*). Similar results were obtained for *A. thaliana* but not for *S. cerevisiae*, because some proteins in the yeast are extremely short or divergent (such as Vps51 or Cog1, respectively). The CC annotation represents the number of amino acids predicted to form CC regions (using ncoils; Lupas et al. 1991) in each protein considered in the a3m file.

For the second approach, we performed HMM comparisons by making the HMMs with the alignments of each CATCHR orthogroup including and excluding the CC region. To remove the CC region, we aligned all the human CATCHR sequences against the HMM of each chain of the cryo-electron microscopy reconstruction of the exocyst (PDB code, 5yfp; Mei et al. 2018), localizing more precisely the beginning of the helical bundles. For the identification of clusters in these data sets, we performed a clustering network analysis using gephi (Bastian et al. 2009) and the modularity algorithm (Blondel et al. 2008) to identify the different clusters. The raw data obtained in both approaches and the MSA for building the HMM are provided in supplementary information data, Supplementary Material online.

### Protein Structure Selection and Comparison

We created a list of proteins containing the CATCHR proteins of each orthogroup, and related and nonoverlapping proteins whose structures were downloaded from the PDB database (descriptions in supplementary table 2, Supplementary Material online). Additionally, we created another set of proteins to compare the N- and C-terminal fragments of the CATCHR subunits that are described in supplementary table 2, Supplementary Material online. We used visual inspection to classify and divide the PDB files of this subset into N- and C-terminal fragments by considering previously described subdomains A–B and C–D–E (Chen et al. 2017). All-versus-all comparisons were calculated with both sets using a new version of the MOMA program to evaluate the structural similarities of these proteins, using the script “MOMA2_pw.py” to calculate flexible pairwise alignments (Gutiérrez et al. 2016; software available at https://hub.docker.com/r/fggutierrez2018/moma2). The scores obtained from these superpositions were collected to create an asymmetric heatmap, where the positions below the diagonal show the probability of the similarity reported in the comparison of the secondary structural elements matrices (*B*_score_) based on a distribution of matrix alignments derived from unrelated proteins. Positions above the diagonal include the total number of equivalent residues observed from the flexible superpositions, and the diagonal of the heatmap reports the length of the structures. Finally, figures of structural alignments were generated using the PyMOL program.

## Supplementary Material

Supplementary data are available at *Genome Biology and Evolution* online.

## Supplementary Material

evab125_Supplementary_Data
