# Supplementary material for: Homology and Modular Evolution of CATCHR at the Origin of the Eukaryotic Endomembrane System
Source: Genome Biol Evol. 2021 Jun 1;13(7):evab125. doi: 10.1093/gbe/evab125 (PMC8290106; doi:10.1093/gbe/evab125)
Supplement: evab125_Supplementary_Data [file evab125_supplementary_data.zip › CATCHR_full_SuppInfo.pdf]

Homology and modular evolution of CATCHR at the origin of the eukaryotic endomembrane-system

Carlos Santana-Molina[1*], Fernando Gutierrez[1, 2] and Damien P. Devos[1*]

[1] Centro Andaluz de Biología del Desarrollo, Consejo Superior de Investigaciones Científicas/Universidad Pablo de Olavide/Junta de Andalucía, Seville, Spain.

[2] Departamento de Genética Molecular y Microbiología, Facultad de Ciencias Biológicas, Pontificia Universidad Católica de Chile, Alameda 340, Santiago, Chile.

* Author to whom correspondence should be addressed: csantmol@gmail.com (C.S.-M.) and damienpdevos@gmail.com (D.P.D.)

Supplementary Information Appendix

Supplementary data

Data.zip contains multiple raw data generated in this study. **A)** Raw data of reciprocal searches based on HMM and single proteins starting from the sequences of *Homo sapiens*, *Saccharomyces cerevisiae* and *Arabidopsis thaliana.* **B)** Multiple sequence alignments (MSAs) are divided into MSAs by orthologs (ortholog_MSA) and the merged alignments by cluster (g51, g52, g53 and g54; cluster_MSA). **C)** Raw data of HMM comparisons from the different analyses: starting from sequences of *H. sapiens*, *S. cerevisiae* and *A. thaliana,* and the orthologous data set with coiled-coil and without coiled-coil.

Supplementary tables

Supplementary Table 1. Orthology analyses. Distribution and protein IDs for the CATCHR orthologs detected in this study through the two methods: reciprocal searches based on HMM and single proteins. Note that the final table is a combination of both approaches in comparison with other analyses and potential orthologs described in UniProt. The sheets 2 and 3 contain the raw data for the reciprocal hits in both analyses.

Supplementary Table 2. PDB codes and additional information for the CATCHR structures analyzed in this study.

Supplementary figure legends

Supplementary Figure 1. Structural features of CATCHR orthologs. The orthologs forming each CATCHR are grouped within the squares. Sequences are represent linearly mapping secondary structure and Pfam domains. For the secondary structure prediction, pink bars indicates alpha-helix and cyan bars indicates beta-sheets. The line plot below

indicates structural protein disorder predicted. Sequences are sorted taxonomically: protist, plants, fungi and animals. Note that sets of orthologs have different scales. Taxonomy keys are the following: TOXGV, *Toxoplasma gondii*; PLAF7, *Plasmodium falciparum*; THEAN, *Theileria annulata*; BABBO, *Babesia bovis*; CRYPI, *Cryptosporidium parvum*; LEIMA, *Leishmania major*; TRYB2, *Trypanosoma brucei*; BODSA, *Bodo saltans*; NAEGR, *Naegleria gruberi*; THAPS, *Thalassiosira pseudonana*; PHYIT, *Phytophthora infestans*; BLAHO, *Blastocystis homonis*; ENTHI, *Entamoeba histolytica*; DICDI, *Dictyostelium discoideum*; GALSU, *Galdieria sulphuraria*; MICCC, *Micromonas commoda*; OSTTA, *Ostreococcus tauri*; PHYPA, *Physcomitrella patens*; ARATH, *Arabidopsis thaliana*; MONBE, *Monosiga brevicollis*; CRYNJ, *Cryptococcus neoformans*; YEAST, *Saccharomyces cerevisiae*; SCHPO, *Schizosaccharomyces pombe*; GIBF5, *Gibberella fujikuroi*; PENRW, *Penicillium rubens*; CAEEL, *Caenorhabditis elegans*; DROME, *Drosophila melanogaster*; DANRE, *Danio rerio*; HUMAN, *Homo sapiens*.

Supplementary Figure 2. A) Clustering network of HMM comparisons between CATCHR proteins considering the coiled-coil region. The HMM were built using the the sequences obtained from the orthology analyses. Nodes are organized and colored by modularity. **B)** Clustering network of g53 and g54 clusters. **C)** Clustering network of the four first hits between CATCHR HMM. Nodes are organized by complexes.

Supplementary figure 3. A) Distribution of the alignment coverage and **B)** e-values of the HMM comparisons between protein from one cluster with the others. The HMM comparisons are those performed from human sequences. Vertical lines indicates the potential end of the coiled-coil of the respective CATCHR protein.

Supplementary Figure 4. Multiple sequence alignments (MSAs) of each CATCHR cluster and mapping of the structural information. MSAs are colored according to Clustal scheme: hydrophobic, blue; positive chaged, red; negative charged, magenta; polar, green; cysteine, pink; glycine, orange; proline, yellow; aromatic, cyan; unconserved, white. Black lines below the alignments represents the length and coverage of the respective PDB structures in the MSA. MSA are provided in **Supplementary Information Data**.

Supplementary Figure 5. Multiple sequence alignments of specific positions generally conserved between the CATCHR domains C-D of all g53 and g54 proteins. The structural alignment of such positions is shown in Figure 4. Positions are numbered according to Tip20 amino acids.

Supplementary Figure 6. A) Structural alignment of g52 (Exo70) and g53/g54 (Sec10/Tip20) proteins. Structural alignment were conducted with pymol. **B)** All versus all structural comparison of CATCHR structures separated in half (A-B and C-D-E domains). The asymmetrical heatmap represents the MOMA structural similarity scores (log_{10}) below the diagonal and the respective length of the alignments above the diagonal. The labels are sorted according to the average of the similarity scores. **C)** Structural alignment of CATCHR domains A-B of g52 (Exo70) and g51 (Exo84) proteins. Structural alignment were conducted with MOMA. Red/orange regions indicates high structural similarity whereas green/blue regions indicate low structural similarity.

Supplementary Figure 7. A) Workflow for the identification of of CATCHR orthologs by reciprocal searches based on HMM and single proteins. **B)** Workflow for the detection of

homology between CATCHR proteins. Two methods employed, one by automatic generation of HMM and the other by the generation of HMM from the sequences obtained in the identification of orthologs.

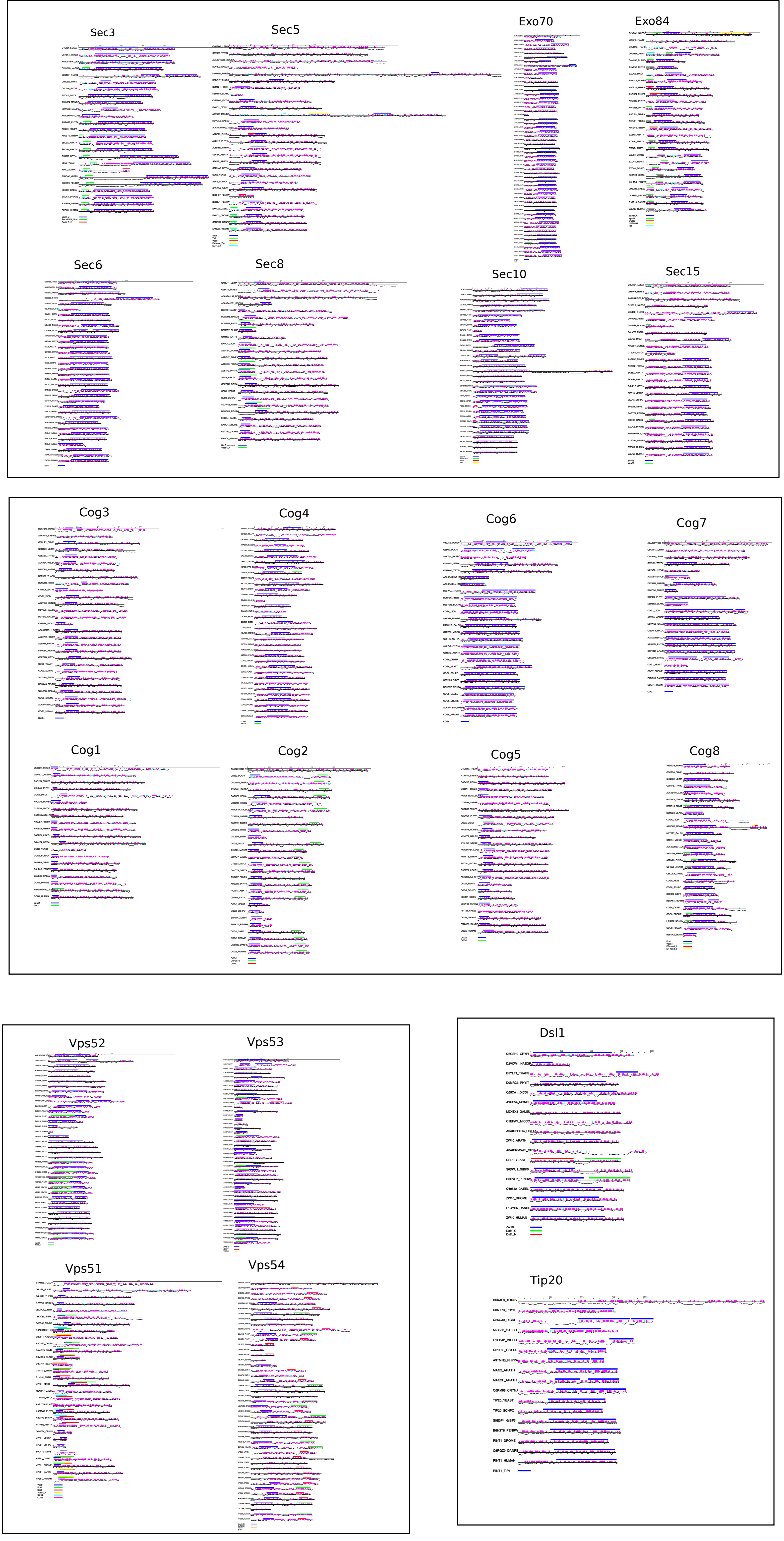

Supplementary figure 1

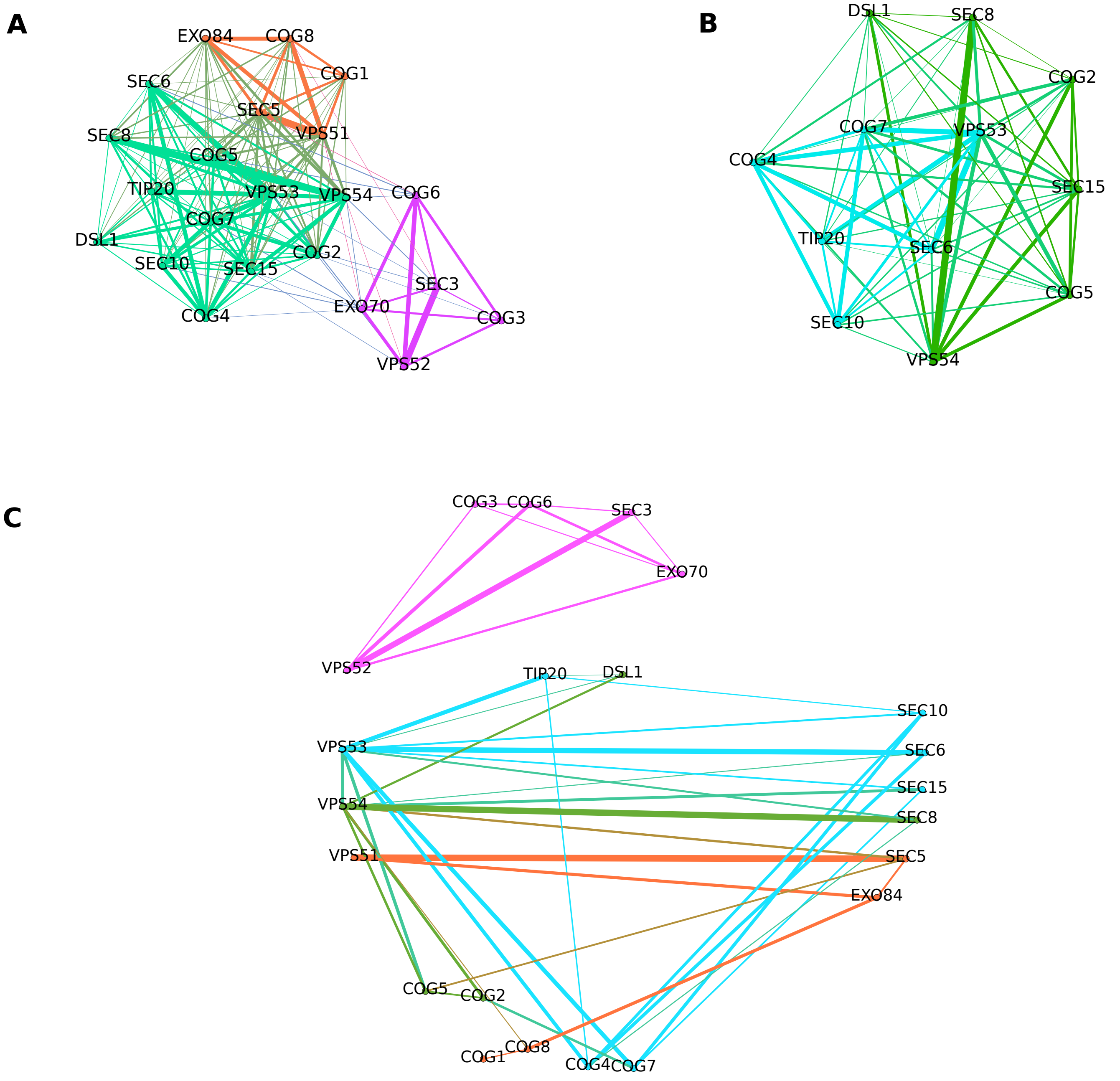

Supplementary Figure 2.

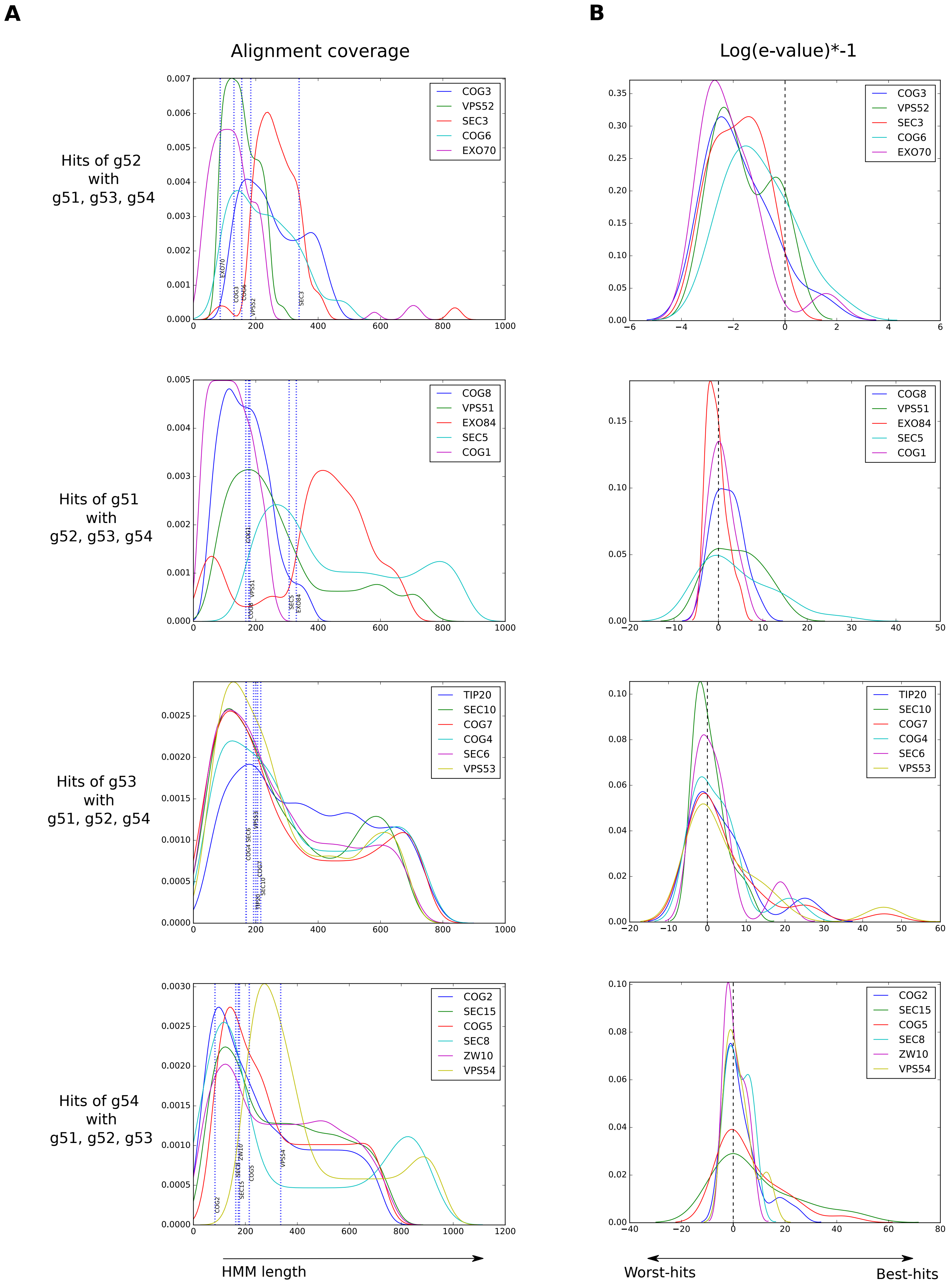

Supplementary Figure 3

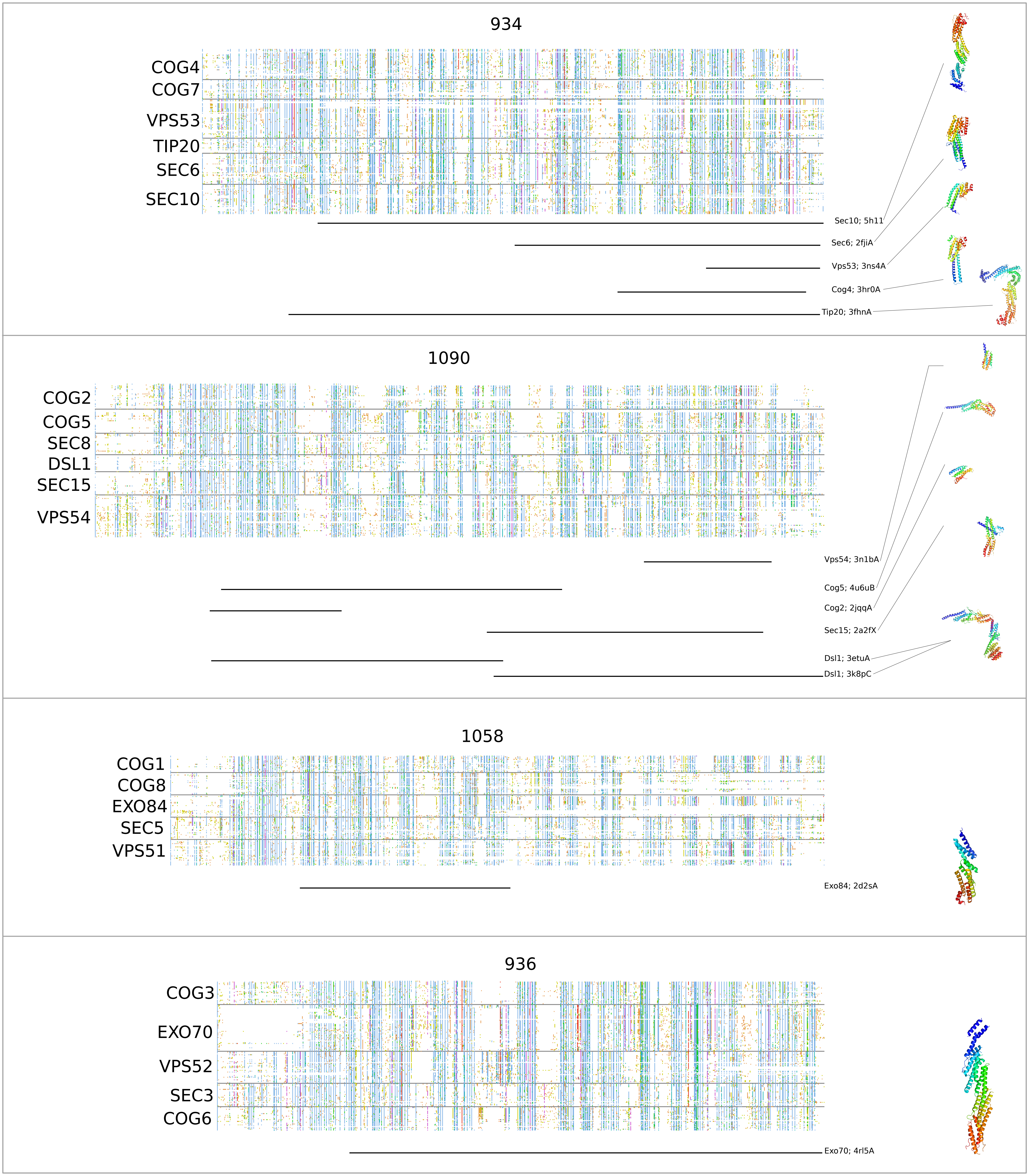

Supplementary Figure 3.

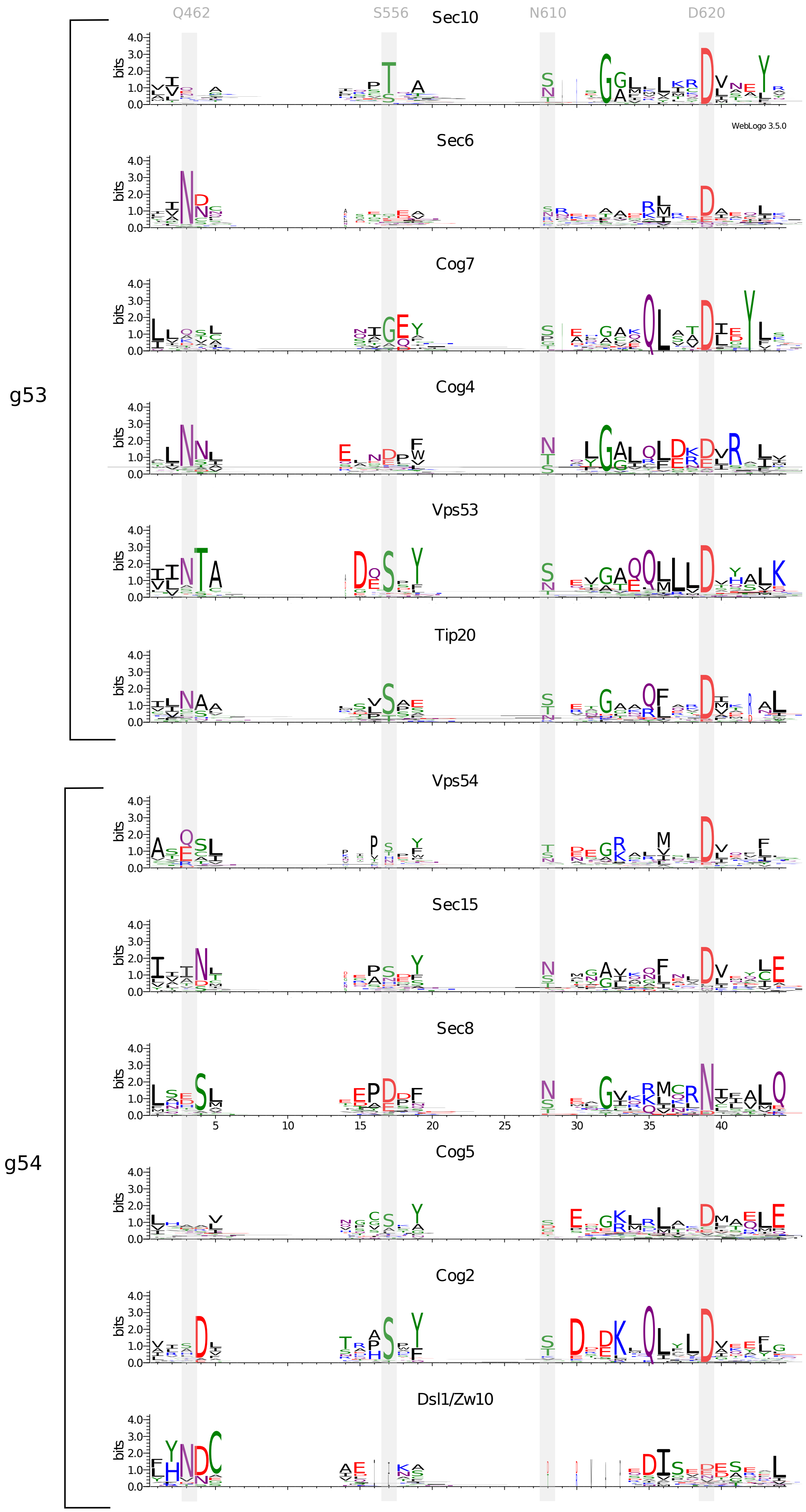

Supplementary Figure 5

A

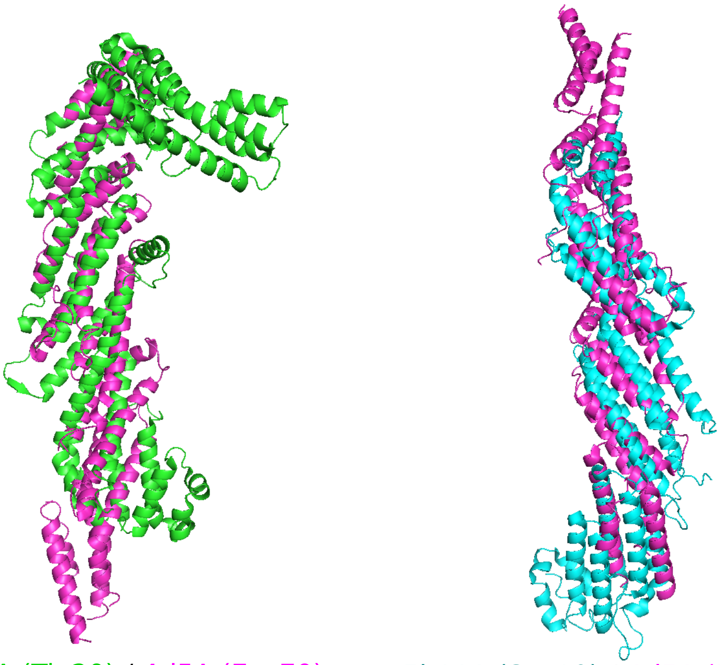

3fhnA (Tip20) / 4rl5A (Exo70)   5h11A (Sec10) / 4rl5A (Exo70)

B

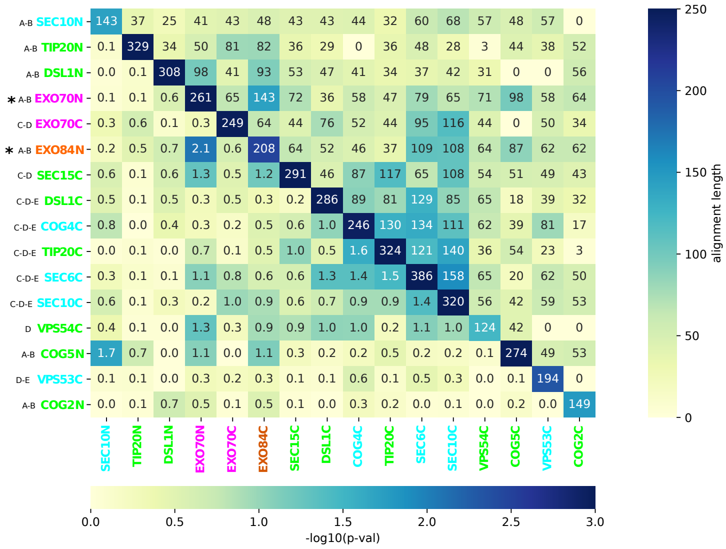

C

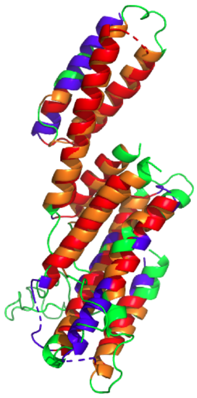

Exo84 (**2d2sA**)
vs.
Exo70 (**4rl5A**)

Supplementary Figure 6

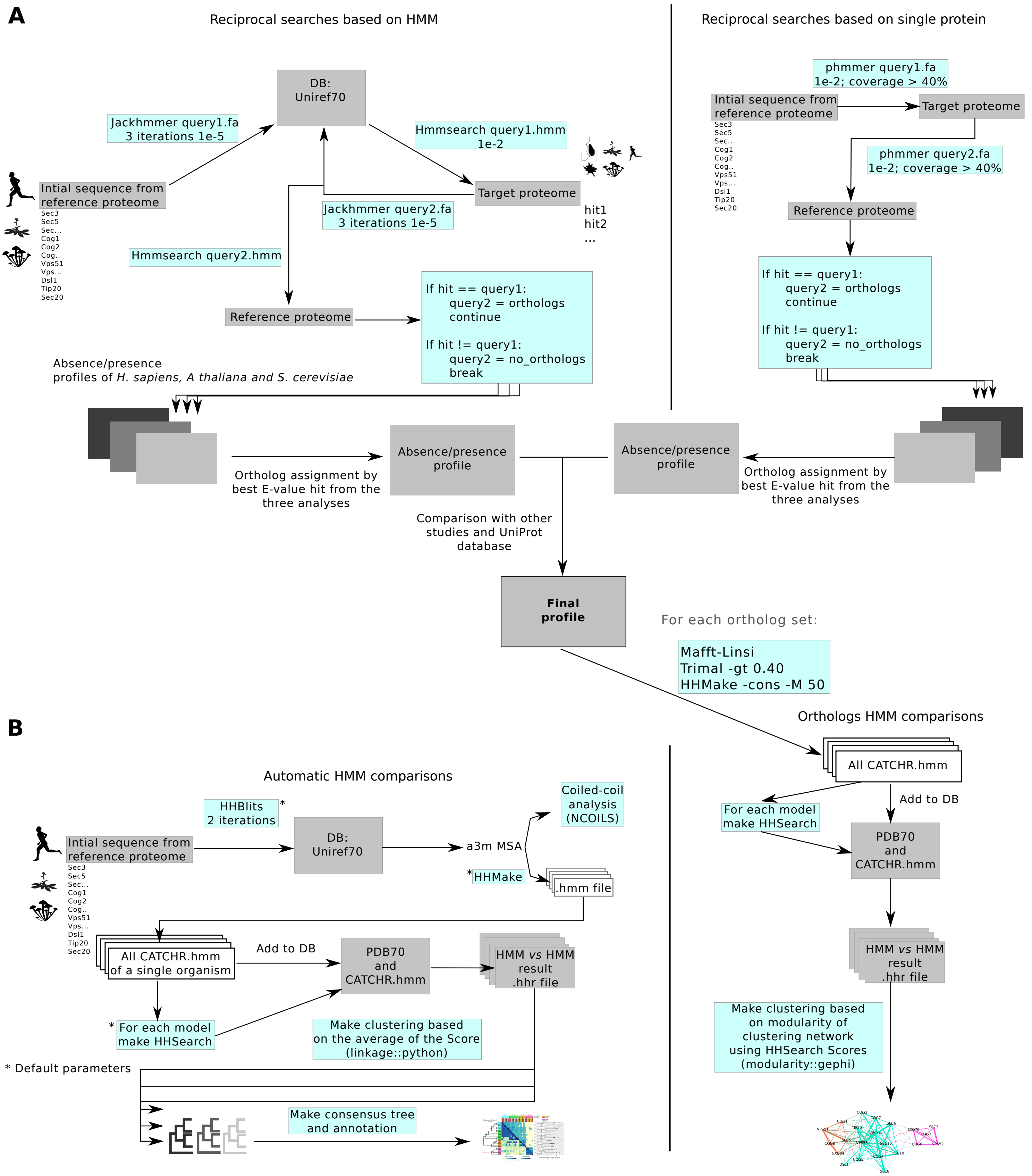

Supplementary Figure 7
